# A systematic review of genetic ancestry as a risk factor for incidence of non-small cell lung cancer in the US

**DOI:** 10.3389/fgene.2023.1141058

**Published:** 2023-04-04

**Authors:** Breanna A. James, Jennie L. Williams, Barbara Nemesure

**Affiliations:** Stony Brook Medicine, Department of Family, Population, and Preventive Medicine, Stony Brook, NY, United States

**Keywords:** lung cancer, genetics, racial disparities, risk factors, non-small cell lung cancer, genetic ancestry, biomarkers

## Abstract

**Background:** Non-Small Cell Lung Cancer (NSCLC), the leading cause of cancer-related death in the United States, is the most diagnosed form of lung cancer. While lung cancer incidence has steadily declined over the last decade, disparities in incidence and mortality rates persist among African American (AA), Caucasian American (CA), and Hispanic American (HA) populations. Researchers continue to explore how genetic ancestry may influence differential outcomes in lung cancer risk and development. The purpose of this evaluation is to highlight experimental research that investigates the differential impact of genetic mutations and ancestry on NSCLC incidence.

**Methods:** This systematic review was conducted using PubMed and Google Scholar search engines. The following key search terms were used to select articles published between 2011 and 2022: “African/European/Latin American Ancestry NSCLC”; “Racial Disparities NSCLC”; “Genetic Mutations NSCLC”; “NSCLC Biomarkers”; “African Americans/Hispanic Americans/Caucasian Americans NSCLC incidence.” Systematic reviews, meta-analyses, and studies outside of the US were excluded. A total of 195 articles were initially identified and after excluding 156 which did not meet eligibility criteria, 38 were included in this investigation.

**Results:** Studies included in this analysis focused on racial/ethnic disparities in the following common genetic mutations observed in NSCLC: KRAS, EGFR, TP53, PIK3CA, ALK Translocations, ROS-1 Rearrangements, STK11, MET, and BRAF. Results across studies varied with respect to absolute differential expression. No significant differences in frequencies of specific genetic mutational profiles were noted between racial/ethnic groups. However, for HAs, lower mutational frequencies in KRAS and STK11 genes were observed. In genetic ancestry level analyses, multiple studies suggest that African ancestry is associated with a higher frequency of EGFR mutations. Conversely, Latin ancestry is associated with TP53 mutations. At the genomic level, several novel predisposing variants associated with African ancestry and increased risk of NSCLC were discovered. Family history among all racial/ethnic groups was also considered a risk factor for NSCLC.

**Conclusion:** Results from racially and ethnically diverse studies can elucidate driving factors that may increase susceptibility and subsequent lung cancer risk across different racial/ethnic groups. Identification of biomarkers that can be used as diagnostic, prognostic, and therapeutic tools may help improve lung cancer survival among high-risk populations.

## 1 Introduction

Lung cancer is the second most diagnosed cancer and leading cause of cancer related death in the United States, with the Non-Small Cell Lung Cancer (NSCLC) subtype constituting 85% of all lung cancer cases (Chen et al., 2014; Siegel et al., 2022). There are known racial and ethnic differences with regard to lung cancer risk, survival, and mortality in African Americans (AAs), Caucasian Americans (CAs), Hispanic Americans (HAs), and Latin Americans (LAs) (Alexander et al., 2016; Siegel et al., 2022). Despite lower smoking prevalence, smoking initiation later in life, and low smoking intensity, AAs experience the highest burden of lung cancer in comparison to CAs (Singh and Jemal, 2017). In contrast, HAs experience the lowest burden of lung cancer (Patel et al., 2013). Although recent studies have shown that genetic variations among AAs, CAs, and HAs play a significant role in NSCLC risk, survival, and mortality, what the nature of the variations are and how they differ in relation to each racial/ethnic group remains unclear (Haiman et al., 2006; Meza et al., 2015; Houston et al., 2018).

Investigation into the role of genetics in racial/ethnic differences relative to NSCLC has uncovered influential factors: the presence of susceptibility alleles, ancestral background, and mutational frequencies of several oncogenic drivers involved in NSCLC carcinogenesis (Gutierrez et al., 2017). The onset of NSCLC is generally caused by genetic mutations that lead to the activation of oncogenic drivers (Gutierrez et al., 2017). However, each histological subtype of NSCLC develops in distinct ways depending on functional changes in the mutant gene (Landi et al., 2021; Mack et al., 2022). Understanding the mechanism of each mutation can be used to characterize the tumor biology of NSCLC, as well as identify correlations between different mutations associated with NSCLC with implications on diseases prognosis and overall survival outcome (Brawley et al., 2021). Genetic mutations have varying consequences as a result of functional changes (silenced, reduced activity, or hyperactivity) that can interrupt normal physiological processes, ultimately contributing to lung tumorigenesis and maintenance of the tumor microenvironment. The most common mutated genes identified in NSCLC include *KRAS*, *EGFR*, *TP53*, *PI3KCA*, *MET*, *BRAF*, *ALK* Translocations, and *STK11*/*LTKB-1* (Ding et al., 2008; Yamaguchi et al., 2013). Although this is not an extensive list of mutations found in NSCLC, many of the studies included in the results analysis section incorporate one or more of these genes in their investigation. A few studies also observed ERBB2 (HER2) (Sholl et al., 2015; Campbell et al., 2016; Leal et al., 2021), AKT (Sholl et al., 2015; Campbell et al., 2016; Leal et al., 2021), *NRAS* (Sholl et al., 2015), *MAP2K1*/*MEK1* (Sholl et al., 2015; Leal et al., 2021), and numerous combinations of co-occurring mutations (Arbour et al., 2018).

The mutational frequency of the aforementioned genes provides valuable information regarding genetic mutations/alterations that are present in the tumor microenvironment. By comparing the mutational frequencies of genes associated with NSCLC, the data enables researchers to assess if certain oncogenic drivers are mutated more frequently in certain racial/ethnic groups, in addition to investigating whether certain behaviors or other variables (such as age, gender, histological subtype, smoking status, etc.) are directly correlated to certain mutations (Campbell et al., 2016; Palazzo et al., 2019). Another risk factor that recent studies have begun taking into consideration in NSCLC is genetic ancestry. Aside from race and ethnicity, genetic ancestry encompasses more individuals on a larger scale, and considers genetic predispositions, genetic variants, and other associations between different ancestries. Many researchers agree that larger and diverse cohorts are crucial for obtaining a significant quantity of genetic data for these analyses; thus ensuring that results obtained better reflect the entire population, especially those at-risk of developing NSCLC (Jones et al., 2017). These data can highlight novel findings that can be used to assess an individual’s risk of incidence, mortality, and chances of survival (Cannon-Albright et al., 2019).

Several studies have also shown that having a family history of lung cancer increases an individual’s risk of developing the disease, with the highest risk being associated with having a first degree relative who was diagnosed with lung cancer (Lewis et al., 2014; Oak et al., 2020). This factor is independent of race or ethnicity. Despite evidence from previous studies suggesting that ancestry is associated with racial and ethnic differences at the genetic level in NSCLC, it is possible that racial/ethnic differences in genetic mutational frequencies are mainly driven by environmental, social, and lifestyle factors. This review will examine the scientific literature that utilizes genomic analyses to explore how genetic variations and ancestry impact AAs, HAs, and CAs. Such studies suggest promising uses for diagnostic and prognostic biomarkers. Furthermore assessments from a racial/ethnic perspective may be critical for the development of treatment modalities in a diverse patient population.

## 2 Methods

### 2.1 Overview

The literature search for this review was conducted using the PubMed Central database and Google Scholar search engines. Initial key terms used in both search engines included: “African American Ancestry NSCLC,” “European American Ancestry NSCLC,” “Latin American Ancestry NSCLC,” “Racial Disparities NSCLC,” “Genetic Mutations NSCLC,” “NSCLC Biomarkers,” “African American NSCLC Incidence,” “Hispanic American NSCLC Incidence,” and “Caucasian American NSCLC Incidence”. A total of 195 articles were initially identified and subsequently screened to assess whether criteria for inclusion were met. After excluding 156 which did not meet the eligibility criteria, 39 articles were included in this review.

### 2.2 Identification and screening

During the initial search, a filter was applied to exclude articles published prior to 2011. Afterward, any article that included one or more of the key search terms listed previously were selected for screening. This included clinical trials, comparative analyses, systematic reviews, meta-analyses, US-based cohort studies, and international-based cohort studies that incorporated race, ethnicity, sex, and age relative to NSCLC. This search yielded a total of 195 articles.

Moving forward, we identified primary literature, excluding all systematic reviews and other secondary studies. 32 systematic reviews and 17 meta-analyses (N = 49) were excluded. Afterward, we screened the abstracts of the remaining 146 articles to ensure that the paper was focused on NSCLC or one or more of its associated subtypes, leading to the exclusion of 43 articles that did not meet these criteria. Of the remaining 103 articles, 65 articles were further excluded for not meeting the remaining eligibility criteria detailed below.

### 2.3 Review process

**FIGURE 1:**
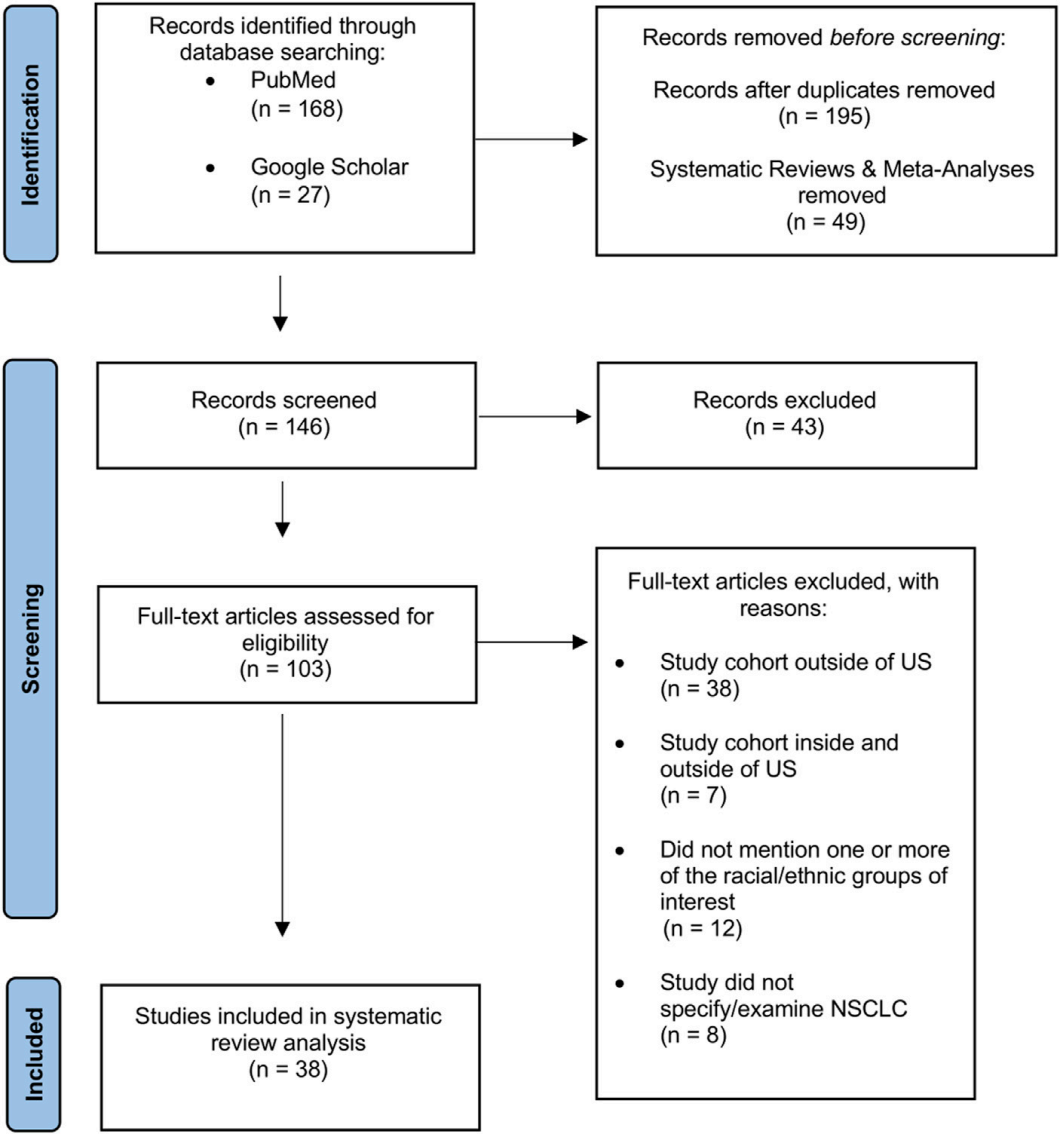
PRISMA flow diagram of article selection for qualitative and quantitative synthesis.

Publications for this systematic review were considered for inclusion based on cohort size, demographic characteristics of each cohort (including age, race/ethnicity, sex), geographic location, ancestral background, mutation analysis, and specific focus on NSCLC and/or associated histological subtypes: Lung Adenocarcinoma (LUAD), Squamous Cell Carcinoma (SCC), and Large Cell Carcinoma (LCC)-N = 47 articles. Publications that mentioned biomarkers, mutation prevalence, and therapeutic methods targeting specific mutations were also included in the discussion section. A PRISMA schematic of the literature screening process can be found in Figure 1.

#### 2.3.1 Cohort size

There are limited number of NSCLC reports in the literature that include a significant number of AAs, Cas, HAs, and LAs. As a result, no criteria specifying the minimum or maximum number of study subjects in a cohort were imposed when assessing eligibility. Instead, emphasis was placed on general racial/ethnic representation in a cohort, resulting in a varying range of cohort sizes observed in different studies (Tables 2, 3).

#### 2.3.2 Demographic characteristics

During the screening phase, relevant articles with study cohorts that incorporated one or more racial/ethnic groups of interest (African Americans, Hispanic Americans, Latin Americans, and Caucasian Americans) were included in this systematic review. In the context of this review, Latin Americans and Hispanic Americans are considered two distinct ethnic groups. This was to ensure that sufficient evidence for each racial and ethnic group was included, even if all 4 groups were not mentioned or incorporated into a given study. For studies focused on genetic ancestry, this criterion was also used for ancestral groups of interest (African, European, and Latin American ancestries). Furthermore, studies that examined age, sex, and smoking history: Current Smoker (CS), Former Smoker (FS), and Never Smoker (NS) as covariates were also included to assess potential relationships between each covariate and genetic factors highlighted in this review.

#### 2.3.3 Geographic location

This review article focuses specifically on AA, HA, LA, and CA populations. Studies conducted anywhere other than the US or that included individuals outside of the US in addition to a US based cohort were excluded. All genomic analyses and genetic ancestry studies were conducted in US-based cohorts; patients currently living in the United States. There were no specific criteria for regions in the United States. Supplementary Table S1 highlights a summary of each study design, the iterature results and racial/ethnic demographic characteristics for each cohort.

#### 2.3.4 NSCLC histological sub-types

There are two general types of lung cancer: Small Cell Lung Cancer and Non-Small Cell Lung Cancer. This review article focuses on NSCLC since it is the most diagnosed subtype of lung cancer in the United States. Articles were excluded if the type of lung cancer was not specified. Additionally, NSCLC is further categorized into 3 subtypes: Lung Adenocarcinoma (LUAD), Squamous Cell Carcinoma (SCC), and Large Cell Carcinoma (LCC). Studies were included regardless of whether their analyses focused on NSCLC in general, or were subtype specific. Articles were also included if genomic analyses were focused specifically on one or more subtypes of NSCLC (Tables 1, 2).

**TABLE 1:** Mutation prevalence of KRAS, EGFR, and TP53 in NSCLC by race.

Study	Muttion status	NSCLC subtype				Sex		Race			Mutation frequency (%)		
First author, year	Gene	LUAD	SCC	LCC	(NOS)	Male	Female	AA	CA	HA/LA	AA	CA	HA/LA
Yamaguchi et al. (2013)	KRAS	65	0	—	6	0	49	3	67	1	4.23%	94.40%	1.41%
Yamaguchi et al. (2013)	EGFR	84	0	—	2	0	64	4	50	1	4.65%	58.10%	1.16%
Campbell et al. (2017)	KRAS	319	142	14	10	236	273	245	264	—	34%	34%	—
Campbell et al. (2017)	EGFR	319	142	14	10	236	273	245	264	—	15%	12%	—
Campbell et al. (2017)	TP53	319	142	14	10	236	273	245	264	—	56%	65%	—
Lusk et al. (2019a)	KRAS	128	49	-	16	78	115	193	—	—	22%	—	—
Lusk et al. (2019b)	EGFR	128	49	-	16	78	115	193	—	—	11%	—	—
Lusk et al. (2019a)	TP53	128	49	-	16	78	115	193	—	—	14%	—	—
Reinersman et al. (2011)	KRAS	—	—	—	597	—	—	121	476	—	17%	26%	—
Reinersman et al. (2011)	EGFR	—	—	—	597	—	—	121	476	—	19%	13%	—
Sholl et al. (2015)	KRAS*	733	—	—	—	99	151	9	226	—	16%*	27%*	—
Sholl et al. (2015)	EGFR*	733	—	—	—	167	58	14	174	—	25%*	21%*	—
Araujo et al. (2015a)	KRAS	—	142	—	240	208	174	99	283	—	16.20%	18.00%	—
Araujo et al. (2015b)	EGFR	—	142	—	240	208	174	99	283	—	5.10%	6.00%	—
Steuer et al. (2016)	KRAS*	1007	—	—	—	376	550	60	838	28	17%*	27%*	4%
Steuer et al. (2016)	EGFR	1007	—	—	—	376	550	60	838	28	22%	20%	48%
Bauml et al. (2013)	KRAS	88	5	2	—	32	73	13	86	1	25.00%	30.60%	50%
Bauml et al. (2013)	EGFR*	405	46	9	1	210	303	67	399	3	4.80%	13.70%	0%
Vigneswaran et al. (2016)	KRAS	289	—	—	—	116	173	73	187	—	27%	69%	—
Vigneswaran et al. (2016)	EGFR	289	—	—	—	116	173	73	187	—	39%	42%	—
Bollig-Fischer et al. (2015)	TP53	92	29	—	16	80	57	137	335	—	1%	3%	—

*Indicates statistical significance (*p* < 0.05).

**TABLE 2:** Mutation prevalence of other detected genetic mutations in NSCLC by race.

Study	Mutation status	NSCLC subtype				Sex	Race			Mutation frequency (%) (/%)		
		Total (N=)				Total (N=)	Total (N=)					
First author, year	Gene	LUAD	SCC	LCC	NOS	Male/female	AA	CA	HA or LA	AA	CA	HA or LA
Arauz et. al (2020)	STK11*	36	39	—	3	64	82	—	—	15.0%	—	—
						18						
	PIK3CG*	36	39	—	3	64	82	—	—	13.0%	—	—
						18						
	RB1*	36	39	—	3	64	82	—	—	11.0%	—	—
						18						
	CDKN2A*	36	39	—	3	64	82	—	—	10.0%	—	—
						18						
Kytola et. al. (2017)	SPTA1	—	—	—	90	51	58	356	8	37.0%	—	—
						39						
	LRP1B	—	—	—	90	51	58	356	8	47.8%	—	—
						39						
Villaruz et. al. (2015)	BRAF	733	—	—	—	293	1	14	—	6.0%	88.0%	—
						440						
	ALK translocations	733	—	—	—	293	1	50	—	2.0%	94.0%	—
						733						
Ilie et. al. (2013)	BRAFV600E	450	—	—	—	292	—	450	—	—	5.0%	—
						158						
Al-Ahmadi et. al. (2021)	CDKN2A*	253	24	3	—	139	72	208	1	25.0%	21.1%	0%
						156						
	STK11*	253	24	3	—	139	72	208	1	20.8%	16.8%	0%
						156						
	PBRM1*	253	24	3	—	139	72	208	1	11.1%	1.9%	0%
						156						
	SETD2*	253	24	3	—	139	72	208	1	5.6%	0.5%	0%
						156						
	TSC2*	253	24	3	—	139	72	208	1	4.2%	0%	0%
						156						
	FBXW7*	253	24	3	—	139	72	208	1	4.2%	0.5%	0%
						156						
	ALK/EML*	253	24	3	—	139	72	208	1	5.6%	0.5%	0%
						156						
Vigneswaran et. al. (2016)	ALK Translocations	9	0	0	1	5	0	10	—	0%	91%	—
						9						
	ROS1 Rearrangements	1	0	0	0	0	0	1	—	0%	100%	—
						1						
	RET	13	2	0	0	9	5	9	—	31%	56%	—
						7						
	ERBB2	5	0	0	0	3	1	3	—	20%	60%	—
						2						
	PIK3CA	12	7	0	0	14	4	13	—	21%	68%	—
						5						
	MET	6	2	1	1	7	3	4	—	27%	36%	—
						4						

*Indicates statistical significance (*p* < 0.05).

#### 2.3.5 Mutation analyses

After conducting a preliminary search on PubMed, the most prominent genetic mutations that were associated with and implicated in NSCLC were identified as the following: *KRAS, EGFR, TP53, PIK3CA, MET, BRAF, ALK* Translocations, and *STK11*. Studies that investigated one or more of these genes relative to NSCLC at the racial and ethnic level were included in this review. This review simply highlights genes that were frequently mentioned and were the subject of genomic analyses and is not an exhaustive list of each type of mutation discovered in NSCLC. Other genes mentioned in the results also included *ERBB2, NRAS, MAP2K1/MEK1,* and *AKT*. However, these were not considered common mutations associated with NSCLC. Importantly, articles that also discussed potential diagnostic and prognostic biomarkers and those using mutational profiling to enhance existing treatment methods were also incorporated in the *Discussion* section.

## 3 Results

The studies reviewed in this investigation were organized into one of the following categories: gene mutations in NSCLC, genetic Ancestry and family history of NSCLC, and genomic landscape of NSCLC in African, Hispanic, and Caucasian Americans.

### 3.1 Genetic mutations in NSCLC

Studies that compared somatic mutations among one or more racial/ethnic groups or examined the mutational frequencies of somatic mutations in NSCLC in AA, HA, LA, and CA individuals were selected to investigate whether significant differences exist in mutational profiles of oncogenic drivers associated with NSCLC among different racial/ethnic populations. Since most studies examined the mutational profile of more than one gene, this section groups the results of each study associated with each gene (Tables 1, 2).

#### 3.1.1 KRAS

KRAS is the most common oncogenic driver associated with NSCLC incidence, and a frequent gene of interest that is often selected for genotyping analyses. KRAS is a signaling transducer protein that is activated in numerous cell signaling pathways which function to regulate cell growth, cell proliferation, and apoptosis; inclusive of the MAPK pathway (McCubrey et al., 2007; Degirmenci et al., 2020). Genetic alterations in the genes associated with the MAPK pathway may lead to an increase in the expression of signaling transducer proteins and growth factors, leading to tumorigenesis due to uncontrolled cell proliferation, growth, and downregulation or complete inhibition of genes responsible for activating proteins that are responsible for initiating apoptosis (Wang et al., 2019). This has been established to be true for NSCLC patients harboring KRAS mutations. Overall, the mutational frequency of KRAS was consistently higher than other genetic alterations in NSCLC patients across each study in the present review (Table 1). However, differences were negligible at the racial/ethnic level, and significant associations with NSCLC risk and incidence were only found in relation to smoking status, gender, and age.

Campbell et al. (2017) compared the frequency of somatic mutations in AA and CA patients to determine if there was a difference in the frequency of oncogenic drivers between both groups (McCubrey et al., 2007). Results of this study suggested that KRAS mutations were not significantly different between AAs and CAs. However, in another study, Reinersman et al. (2011) discovered the mutational frequency of KRAS was statistically significant (*p* = 0.04). Mutational frequency was higher in CAs (26%) compared to AAs (17%). In addition, Yamaguchi et al. (2013) published similar results with a study cohort that included HAs; in which, the percentage difference was higher. Here the mutational frequency of KRAS was found to be 41.6%, 20%, and 0% in CAs, AAs, and HAs, respectively. Overall, the results from each study led to similar findings which suggest that the mutational frequency of KRAS is not significantly different between CAs and AAs, as well as CAs and HAs (Reinersman et al., 2011; Araujo et al., 2015a; Sholl et al., 2015; Lusk et al., 2019a; Wang et al., 2019).

In addition to observing the mutational profile of KRAS in CAs and AAs, Araujo et al. also used histology, age, and gender as covariates alongside mutational frequency to determine if there was any correlation between each covariate and overall survival in AAs and CAs. No significant differences in the mutational frequency of KRAS between CAs (18.0%) and AAs (15.4%) were observed. In addition, none of the covariates (histology, age, and gender) correlated with overall survival. Despite these findings, this study did observe that KRAS mutations were found more frequently in Lung Adenocarcinoma (LUAD), a common histological form of NSCLC, and were more common in current and former smokers (Lusk et al., 2019a). Steuer et al. (2016) also investigated several LUAD cases harboring KRAS mutations. Although statistically significant, the mutations were notably higher in CAs (27%) than in AAs (17%) (Reinersman et al., 2011).

Studies by researchers Bauml et al. (2013) concluded that the frequency of NSCLC cases harboring KRAS mutations in AA, HA, and CA patients were similar to those reported earlier. However, the findings in this study did reveal a potential association between KRAS mutations and gender; mutations were frequently identified in more females compared to males. Also, a correlation was found between history of smoking and harboring the KRAS mutation (*p* < 0.01) (Bauml et al., 2013). However, there was ultimately no significant differences in average cigarette pack years and the number of patients with KRAS mutations (Bauml et al., 2013). Another comparative study examined several different molecular alterations in AA patients with LUAD, which also included KRAS. In contrast to the other analyses, these were the only results to suggest that KRAS was the most frequently mutated gene in the AA cohort, however, this was not significantly different across racial and ethnic groups (Rodriguez et al., 2019). Additionally, although no correlation between KRAS mutations and survival outcome in AAs was observed, Rodriguez et al. observed worse survival outcomes in AAs than in CAs (Cox and Der, 2010).

#### 3.1.2 EGFR

Epidermal Growth Factor Receptor (*EGFR*) is another oncogenic driver found in NSCLC cases. In addition, a growth factor receptor which regulates cell growth in various cell-signaling pathways, including the MAPK signaling pathway, was also noted (Rodriguez et al., 2019). Previous studies have identified several variants of mutated *EGFR* in patients diagnosed with NSCLC, including *EGFR* exon 20 insertions, *EGFR* exon 19 deletions, and *EGFR* L858R point mutation in exon 21. Although this is not an exhaustive list, these are the most common variants of the *EGFR* mutation that also have clinical significance for NSCLC treatment. The *EGFR* exon 20 insertion variant is known to be resistant to *EGFR* Tyrosine-Kinase Inhibitors (TKIs), a therapeutic agent used to treat patients harboring *EGFR* mutations. Harboring this mutation results in low sensitivity and diminished response to TKIs treatment (Bauml et al., 2013; Steuer et al., 2016; Rodriguez et al., 2019). In contrast, EGFR exon 19 deletions and EGFR L858R point mutations are EGFR-TKI sensitive, and findings suggest that patients harboring these specific EGFR variants have an improved survival outcome (Cox and Der, 2010; Arcila et al., 2013; Steuer et al., 2016; Leal et al., 2021). Most of the studies included in this systematic review make a distinction between each EGFR variant in molecular analyses, although a few studies only reported data on the general EGFR gene (Table 2).

Campbell et al. (2017), analyzed the frequency of both *KRAS* and *EGFR* mutations in AAs and CAs. Similar to the mutational frequency of *KRAS* in this study, there was no significant difference in *EGFR* mutational frequency among both groups. Yet another analysis of *EGFR* mutational frequency in AAs and CAs confirmed no significant differences in both the type of mutant variant (*p* = 0.17) and the overall mutational frequency (*p* = 0.53) in AAs and CAs (Campbell et al., 2016). Multiple studies obtained similar results, and most of the mutations were found in the LUAD subtype and were more common in females regardless of race/ethnicity and in those who were never-smokers (Okabe et al., 2007; Wu et al., 2008; Rosell et al., 2009; Reinersman et al., 2011; Wu et al., 2018; Lusk et al., 2019a; Wang et al., 2019; Degirmenci et al., 2020).

Interestingly, in terms of survival outcome, Araujo et al. also observed better overall survival outcomes in AAs harboring EGFR mutations in comparison to CAs (*p* = 0.067) (Lusk et al., 2019a). However, in a study conducted by Cheng et al. (2020) focusing solely on differences in wild-type EGFR versus EGFR mutations and survival outcome, the opposite was observed in NSCLC patients harboring EGFR mutations. The study population in this study included AA and non-AA cohorts, no further breakdown for racial and ethnic demographics of the non-AA population were provided. The 2-year survival rate of AAs was significantly lower than in non-AAs: 33% versus 61% respectively. Even after the introduction of EGFR-TKIs to treat EGFR-mutant positive NSCLC cases in each group, the 2-year survival rate remained significantly lower in AAs. Additionally, there were no significant differences in the survival rate of AA and non-AA NSCLC patients who retained the wild-type EGFR phenotype (Okabe et al., 2007).

#### 3.1.3 TP53

Tumor protein p53 (*TP53*) gene is responsible for cell cycle regulation, mediating cell proliferation, and inducing apoptosis in damaged cells that are not successfully recovered *via* cell repair mechanisms. Characterized as a tumor suppressor gene, *TP53* is critical for inhibition of tumor growth. *TP53* transitions from a proto-oncogene to an oncogenic driver when a mutation causing either reduced function or complete silencing of the gene occurs, leading to the loss of a crucial cell control mechanism, ultimately promoting tumorigenesis (Ding et al., 2008; Cote et al., 2011). Mutations in *TP53* occur frequently in several cancer types, including NSCLC (Cote et al., 2011; Bollig-Fischer et al., 2015; Arbour et al., 2018; Arauz et al., 2020; Cheng et al., 2020; Huang et al., 2020; Fan et al., 2022).

Even though mutant *TP53* has been identified in NSCLC, there were few studies that analyzed the mutational frequency of *TP53* at the racial/ethnic level among AAs, CAs, and HAs. Non-etheless, from these few studies there are significant findings worth highlighting (Table 2). In studies that analyzed TP53 as a mutually exclusive mutation, the results suggest that presence of *TP53* mutations in LUAD tumors are associated with NSCLC incidence at an earlier age, as well as a general significant increase in the mutational frequency of *TP53* in AAs compared to other genetic mutations (Bollig-Fischer et al., 2015; Arbour et al., 2018; Arauz et al., 2020). However, only one study suggested that the mutational frequency of *TP53* was higher in an AA subgroup compared to CAs (Arbour et al., 2018), while another found no difference among either group (Fan et al., 2022). In a study conducted by Arbour et al. (2018), co-occurring mutations in *TP53* and *KRAS* were not significantly associated with survival, although *KRAS/TP53* mutant NSCLC was the most common co-occurring mutation identified in this study cohort (Bollig-Fischer et al., 2015). Another study also examined the co-occurring mutational frequencies of *TP53* in conjunction with several other genes: *KDR*, *SMO*, and *CDKN2A*, finding no significant differences in any of the mutant gene combinations in AA patients (approximately 1.8% mutation rate of each co-occurring mutation) (Arbour et al., 2018).

#### 3.1.4 Other mutations

Several studies also examined the mutational frequency of other mutations identified in NSCLC including *ALK* rearrangements, *ROS-1* Rearrangements, *ERBB2*, *BRAF* (*BRAF* V600E) (Ilie et al., 2013; Villaruz et al., 2015; Reckamp et al., 2021), *STK11* (also known as *LTKB1*), *MET* (Krishnaswamy et al., 2009; Kytola et al., 2017), and *PIK3CA*. In the cohort-based studies evaluated for this review, the rate of these genetic alterations was small and generally non-significant, never exceeding 10% in any racial/ethnic group compared to *EGFR*, *KRAS* and *TP53* (McCubrey et al., 2007; Okabe et al., 2007; Wu et al., 2008; Rosell et al., 2009; Reinersman et al., 2011; Cancer Genome Atlas Research Network, 2012; Bauml et al., 2013; Araujo et al., 2015a; Sholl et al., 2015; Campbell et al., 2016; Steuer et al., 2016; Wu et al., 2018; Lusk et al., 2019a; Wang et al., 2019; Degirmenci et al., 2020). However, several of these mutations were associated with smoking status and gender, suggesting that other differences in patient characteristics may be associated with increased likelihood of developing a mutation in comparison to weak associations between race and mutational frequency alone. This is highlighted in the literature review summary table (Supplementary Table S1).

An exception to the above finding is that of a study which observed a 33.3% mutational frequency rate of *ALK* rearrangements in HA. However, a total of nine HA patients were included in this study cohort and *ALK* rearrangements were only identified in three of these individuals (Yamaguchi et al., 2013). Furthermore, statistical evidence from another study comparing the mutational frequencies of several different mutations in AA and CA NSCLC patients (inclusive of ALK rearrangements, PBRM1, SETD2, TSC2, and FBXW7) suggests that the frequency of these mutations in AAs is higher than in CAs. However, the difference in frequency of ALK rearrangements in AAs and CAs was statistically significant: 5.6% and 0.5% respectively (*p* = 0.005) (Al-Ahmadi et al., 2021). These findings emphasize the need for adequate sample size within research studies.

Altogether, there is no indication of any significant differences in the mutational frequency of *ALK* rearrangements at the racial/ethnic level. However, several studies suggest that the frequency of *ALK* rearrangements is higher in never-smokers. In one study, although findings did not reflect any racial/ethnic differences, 61% of *ALK* rearrangements were found in never-smokers (Vigneswaran et al., 2016). Gill et al. found a similar association between ever-smoking and the *STK11*/*LTKB1*, in which a similar mutational frequency of *STK11*/*LTKB1*was found among AAs and CAs. However, these mutations were only found in female who were current and former smokers (Gill et al., 2011).

### 3.2 Genetic ancestry and family history of NSCLC

Studies that compared germline mutations, Single Nucleotide Polymorphisms (SNPs), and other variants at the population-level, in addition to examining the frequency of these variants and their associations with NSCLC incidence, mortality, and overall survival outcome were selected to investigate whether ancestral background (African, European, and Latin American Ancestries) plays a role in observed genetic differences among racial/ethnic populations and as a result, contribute to the socioeconomic and environmental effects that are associated with individuals at high risk for NSCLC incidence and larger NSCLC tumor burden. Few studies also considered population admixture, as many individuals are known to have more than one ancestral background. Genetic ancestry and family history of NSCLC may have a profound influence on potential risk variants that are present in different populations and individuals (Table 3).

**TABLE 3:** Genetic variants detected in genome-wide association studies (GWAS) implicated in NSCLC.

References	Genetic variants	Chromosome location	Gene	Associations	Ancestry
First author, year	SNPs				
Jones et al. (2019)	rs336958*	5q14.3	HNF1B	Increased NSCLC risk	AFR
	rs7186207*	16q22.2	DHODH	Reduced risk of NSCLC	AFR
	rs11658063*	17q12	HAPLN1	Reduced risk of NSCLC	AFR
	rs17486278*	15q25.1	CHRNA3-CHRNB4	Sex specific association in females risk of NSCLC incidence in ES	AFR
	rs7486184*	12q21.32	KITLG	Lung cancer risk in females	AFR
Zanetti et al. (2016)	rs2036527	15q25.1	CHRNA5	Lung cancer risk	AFR
	rs2853677	5p15.33	TERT	Lung cancer risk	AFR
	rs55781567*	15q25	CHRNA5	Lung cancer susceptibility	AFR
	rs3019885*	15q25	CHRNA5	Lung cancer susceptibility	AFR
	rs6580649*	15q25	CHRNA5	Lung cancer susceptibility	AFR
	rs1293936	6q25.1	ESR1	Former smokers and lung cancer susceptibility	AFR
Ji et al. (2020)	rs56009889*	11q22.23	ATM	Lung cancer susceptibility specifically in LUAD, and significantly associated with females	EUR
	rs150665432*	11q22.24	KIAA0930	Lung cancer susceptibility	EUR
	rs61816761*	11q22.25	FLG	Lung cancer susceptibility	EUR

*Indicates statistical significance (*p* < 0.05).

#### 3.2.1 African ancestry

Genetic ancestry level analyses conducted in Genome-Wide Association Studies (GWAS) have successfully identified differences in NSCLC risk, incidence, and survival across ancestral groups (Zanetti et al., 2016; Jones et al., 2019; Schenk et al., 2020; Sinha et al., 2020; Adib et al., 2022). Sinha et al. (2020) compared the frequency of copy number mutations and homologous recombination. The results suggested that genomic instability in AAs with African Ancestry was greater than that of CAs with European ancestry, which is supported by the significantly higher frequency of homologous recombination and structural variants found in AAs and African Ancestry respectively, in contrast to CAs. Another study observed similar results in which AAs diagnosed with LUAD had a higher somatic mutation burden than CAs, Conversely, no significant differences were seen with the SCC histology. Based on the ancestral composition of this study cohort (including admixture), the findings of Schenk et al. (2020) suggest that genetic ancestry potentially influences somatic alterations in LUAD exclusively, especially in AAs (Jones et al., 2019).

Several studies also revealed novel gene variants potentially implicated in NSCLC exclusively with African Ancestry, that were never detected previously in other ancestry groups (Jones et al., 2019; Oak et al., 2020; Sinha et al., 2020). This included a novel association between *BRCA2* and SCC incidence (histological subtype of NSCLC) (Oak et al., 2020). A few of these gene variants were also associated with overall lung cancer risk, and predisposition of NSCLC including: rs33658 (*HNF1B* gene in chromosome 5q14.3) associated with increased lung risk, rs7186207 (*DHODH* gene in chromosome 16q22.2) significantly associated with lower lung cancer risk, and rs11658063 (*HAPLN1* gene in chromosome 17q12) associated with lower NSCLC risk (Jones et al., 2019).

A GWAS study conducted by Zanetti et al. (2016) also discovered two novel loci associations with lung cancer susceptibility in African Americans, including: rs2036527 (*CHRNA5* gene in chromosome 15q25.1) and rs2853677 (*TERT* in chromosome 5p15.33) that was specifically associated with susceptibility for LUAD histology. There was no evidence of associations between any of these novel gene variants and prognosis/overall survival outcome in NSCLC. Overall, findings suggest that genetic ancestry alone is not a significant predictor of lung cancer risk, incidence, nor survival (Araujo et al., 2015b; Zanetti et al., 2016; Jones et al., 2018; Jones et al., 2019; Mitchell et al., 2019; Schenk et al., 2020; Adib et al., 2022).

#### 3.2.2 European ancestry

Genomic analyses of genetic variants and SNPs associated with NSCLC risk, incidence, and survival were initially discovered and defined in European ancestry populations. Findings from these studies have supported genetic ancestry-level analyses, revealing similarities, differences, and novel discoveries in variants between European, African, and Latin American Ancestry populations. The number of genomic studies and the size of cohorts in this ancestral group far outnumbered those for AAs, HAs, and LAs. Thus, our present understanding of NSCLC risk, incidence, and survival is based on these early studies which were not inclusive of other ancestries. Several genome-wide studies have found various different genetic variants associated with increased lung cancer risk, mortality, and survival (Orloff et al., 2012; Saeed et al., 2012; Zhou et al., 2017; Byun et al., 2018; Li et al., 2018; Ji et al., 2020; Zingone et al., 2021). A study conducted recently by Ji et al. (2020) identified several driving germline mutation variants within European ancestry that were associated with an increase in lung cancer risk: rs56009889, rs150665432, and rs61816761 (*p* < 5.0 × 10^−8^). Another study identified a predisposing fusion gene known as KANSARL associated with lung cancer and has only been characterized in European ancestry studies. Gene fusions may also occur during tumorigenesis, and genetic ancestry studies have also examined how this specific genetic alteration impacts risk and mortality in European ancestry groups (Ji et al., 2020). Furthermore, smoking behavior also plays a critical role in the changes observed in some patients that develop NSCLC. Li et al. (2018) explored the relationship between the gene-environment interactions between smoking and genetic mutations among a European descent population, also identifying two novel SNPs: rs6441286 and rs17723637, that were significantly associated with overall lung cancer risk (*p* < 3.5 × 10^−7^) (Centers for Disease Control and Prevention, 2019).

#### 3.2.3 Latin American and Native American/Alaska Native ancestry

The literature search did not yield a significant number of studies reporting genetic ancestry-level analyses within Latin and Native American populations. According to a genomic ancestry level study conducted by Oak et al. (2020), the cohort population representing Native/Latin American ancestry only comprised 0.4% of the total study population, and there were no significant findings in this study suggesting any significant associations between NSCLC risk, incidence, mortality, or survival and Native/Latin American ancestry. In the US, HAs have the lowest rate of NSCLC incidence and are generally considered to have minimal risk of lung cancer (Gimbrone et al., 2017). In one study focusing on ancestry markers in Latin American ancestry populations, Gimbrone et al. (2017) found that in LUAD cases, there was a significant difference between the mutational frequency of EGFR in Hispanic/Latin American patients compared to non-Hispanic/Latin American groups: 31% and 17% respectively (*p* < 0.001) (Ten Haaf et al., 2017). This study also analyzed KRAS (20% and 38% respectively, *p* = 0.002), STK11 (8% and 16% respectively; *p* = 0.65) and TP53 (46% and 40% respectively, *p* = 0.355), although none of these differences were significant. Moreover, the results from this study suggested that Hispanic/Latin American ancestry is potentially associated with the rate of TP53 mutations (*p* = 0.009) and may also be associated with the rate of EGFR, KRAS, and STK11 mutational frequencies (Aldrich et al., 2013). Additionally, the rates for Native American/Alaska Native are higher than those for CAs. Specifically, these rates are higher in Alaska, Northern Plains, Pacific Coast, and Southern Plains (Coté et al., 2012) (US PRCDA 2012–2016).

## 4 Discussion

The roles of environment, lifestyle and behavioral habits, and social factors are well characterized as driving factors that increase risk of within the US population. Recently, emphasis has been placed on the significance of population genetics in disease. Population genetics can reveal associations and determinants that increase or lower risk of disease, or influence disease outcome. This has led to an interest in personalized medicine. However, until quite recently, factors such as race, ethnicity and gender were not considered. There are observed differences in NSCLC risk and incidence at the racial and ethnic level that affect survival outcome in NSCLC patients. However, the exact determinants remain unclear due to the complexity of acquiring and assessing the contribution of these risk factors within a diverse patient population. To address this, ongoing NSCLC studies have incorporated genomic analyses and population genetics to explore the role of underlying genetic differences including genetic mutations and ancestry in lung cancer to further our understanding of the contributing factors associated with these racial/ethnic differences in NSCLC. Although genetic variations exist, the findings from this systematic literature analysis suggest that changes at the genetic level are likely activated by gene-environment interactions and that differences in racial/ethnic and ancestral backgrounds alone may have less of an independent impact than previously thought.

### 4.1 Genetic mutational frequencies and genetic ancestry

The studies included in this review were separated into two focus groups: analyses of single and co-occurring somatic gene mutations and mutational frequencies in NSCLC and genome level analyses at the ancestral and familial level. Among the most common genetic alterations identified in NSCLC were *KRAS*, *EGFR*, and *TP53*. Other less significant mutations were noted in the results section. Of these, none were found to have significantly different frequencies in AAs, CAs, nor HAs. With the exception of *EGFR*, somatic gene mutations were least likely to be found in HAs. This is especially true of *KRAS* and *STK11* mutational frequencies which were found to be relatively negligible. This can also be attributed to the low lung cancer incidence rate in HAs. Differences in mutational frequencies were observed relative to the general patient population and histological subtype of NSCLC. *KRAS* mutations were the most common somatic mutations found in NSCLC, followed by *EGFR*. Moreover, mutations in *KRAS*, *EGFR*, *BRAF*, *TP53*, and *STK11* were frequently identified in LUAD patients, while *PIK3CA*, and *TP53* mutations were commonly found in SCC patients (Campbell et al., 2016).

Significant associations were also found among genetic ancestry groups and variants associated with lung cancer risk, incidence, and survival. Previous studies have investigated the potential association between family history and increased risk of developing NSCLC (Haiman et al., 2006; Coté et al., 2012), although the exact impact in comparison to other environmental and lifestyle risk factors remain unknown. Admixture is also important to consider when conducting genetic analyses, as many individuals do not solely identify with one ancestry (Coté et al., 2012). Genetic analyses also revealed associations between NSCLC histological subtypes (LUAD and SCC), demographics (age and sex), and smoking status [former smoker (FS), current smoker (CS), or never smoker (NS)]. The findings suggest that gene-environment interactions collectively drive NSCLC risk/incidence, and race/ethnicity does not appear to have a significant influence on differences in NSCLC incidence as an independent factor. Even though the results from current studies suggest there are no differences in genetic mutations at the racial/ethnic level, available data may not accurately reflect the actual statistics once limitations in cohort design and low AA and HA patient participation in genomic analyses at the racial and ethnic level are considered. Future studies should continue to explore the parameters mentioned previously in racially and ethnically diverse patient cohorts, with efforts to incorporate enough participants to represent each racial and ethnic group equally, to improve comparability of data across studies. Non-etheless, the evidence presented in these genetic analyses strongly suggest that gene-environment interactions collectively play a major role in NSCLC risk and incidence, and may significantly influence differential survival outcomes in AA, CA, LA, and HA NSCLC patients.

### 4.2 Environmental and behavioral risk factors

Risk of NSCLC incidence is driven by environmental and behavioral risk factors that either directly or inadvertently generate favorable conditions for lung tumorigenesis (Haiman et al., 2006; David et al., 2016; Stram et al., 2019). Understanding the implications of these factors in NSCLC incidence, mortality, and survival outcomes at the racial and ethnic population level is paramount to addressing racial disparities that amplify detrimental effects of risk factors as a result. Racial/ethnic cohort studies have attempted to address these differences and help to supplement and diversify sample populations in clinical research. As a result, studies have revealed numerous racial/ethnic health disparities in environmental and behavioral factors associated with risk of NSCLC incidence in addition to disparate effects on AAs, CAs, LAs, and HAs. The following subsections highlight environmental, behavioral, and social factors that may initiate/promote genetic changes related to NSCLC, as well as the clinical value of genetic analyses.

#### 4.2.1 Smoking status and gene-environment interactions

Smoking is considered the main risk factor associated with an increased risk of NSCLC incidence (Aldrich et al., 2019; Parekh et al., 2019). Researchers have closely assessed the relationship between smoking habits and lung cancer, including smoking intensity (measured in cigarettes per day- CPD), age of smoking initiation, time since smoking cessation, and cigarette pack years (Wang et al., 2021). In a recent study, Wang et al. (2021) examined the potential relationship between smoking history and mutational burden in advanced stage NSCLC. Participants were advanced stage NSCLC patients who were categorized based on smoking status (never smokers, former smokers, and current smokers) (Wassenaar et al., 2015). The results suggested that a dose-response relationship relative to smoking status and tumor burden does exist. Furthermore, Wang et al. (2021) observed the mutational rates of EGFR and KRAS subtypes in each group. The findings from the EGFR mutation analysis were statistically significant, suggesting that there is a potential indirect relationship between the frequency of EGFR mutations and cigarette pack years. Interestingly, doubling cigarette pack years was associated with a decrease in risk of developing the EGFR exon 19 mutation. Further research is needed to replicate these genetic analyses, especially in clinically and demographically diverse study cohorts (Wassenaar et al., 2015).

This has led to the observation of many significant differences in risk among racial/ethnic groups associated with smoking. In one of the largest ongoing racial/ethnic cohort studies to date, the Multiethnic Cohort Study (MEC) was designed to investigate any correlation between behavioral patterns and onset of disease across 5 racial/ethnic groups in Hawaii and California, including AAs, LAs, and Whites (Haznadar et al., 2016). Stram et al. (2019) developed a sub-study to explore the effects of smoking habits on the risk of lung cancer incidence, mortality, and survival outcome. The results of this study suggest that AAs had the highest overall incidence rate of lung cancer regardless of smoking status (Blot et al., 2011). Additionally, AAs who identified as current smokers also had the highest estimated excess relative risk (a measure of the rate of lung cancer incidence based on level of smoking exposure) of developing both LUAD and SCC subtypes, with statistically significant differences of risk found in individuals that smoke 10–35 cigarettes per day (CPD) in contrast to Whites, while Latinos had the lowest overall risk and estimated excess relative risk of lung cancer. Some key takeaways include that African Americans tend to smoke less CPD, suggesting lower smoking prevalence. When values were controlled for smoking intensity among current smokers to assess excess relative risk, the same trends persisted (Tanner et al., 2020). In other smoking studies, similar smoking habits and trends in AAs were observed, even though statistics consistently demonstrated that AAs tend to begin smoking later in life, at a lower smoking intensity (less CPD), and shorter smoking duration in comparison to Caucasian Americans (Govindan et al., 2012; Nemesure et al., 2021). What was not accessed and may play a considerable role in lung cancer and the smoking trends of AAs was the type/brand of cigarettes smoked. It has been suggested that filtered, methanol, and low tar cigarettes, which were all more prevalent choices of AA, result in deeper inhales and changes in smoking behaviors resulting in increased consumption of nicotine and other toxic hazards found in cigarettes. Overall, studies examining differences in smoking behavior among racial/ethnic groups have suggested that the type of cigarette/tobacco product used may also influence risk of NSCLC incidence. Smoking frequency and intensity among African American have been lower compared to whites and American Indian and Alaska Natives, but tobacco-caused morbidity and mortality rates are disproportionately higher. The characteristics of smoking in AAs and associations with lung cancer collectively define “The African American Smoking Paradox.” The mechanism(s) behind this phenomenon remain unknown (Alexander et al., 2016).

Further investigations are needed to explore the mechanism of smoking and contribution to NSCLC incidence differences in different histological subtypes, as well as its potential effects on racial and ethnic populations. Despite the dominant role of smoking in NSCLC incidence, smoking as a risk factor is not actively studied in never-smokers. As such, the causal factors of NSCLC incidence in never-smokers has not been fully characterized. Incorporating never-smokers into cohort studies at the racial ethnic level is critical because the overall incidence, mortality, and survival outcome rates of each group are comprised in part by never-smokers (Persky et al., 2013; Gu et al., 2017).

#### 4.2.2 Geographic location

Racial disparities potentially have differential effects on AAs, CAs, and HAs when looking at each racial and ethnic population at a specific geographic location (DeSantis et al., 2016; Basu et al., 2021). Previous studies have already highlighted differences in geographic location that could impact how each racial and ethnic group experiences these disparities depending on where they are located, including an individual’s behavioral habits, lifestyle (diet, exercise), occupation, and socioeconomic status (Hastert et al., 2015; Ellis et al., 2018; Zahnd et al., 2018; Klugman et al., 2020; Sanchez et al., 2020). For example, locations at higher altitudes, rural areas, and urban areas may also potentially influence how HAs, CAs, and AAs are affected by the environmental and behavioral risk factors listed previously (Johnson et al., 2014; Islam et al., 2015; Moore et al., 2017). Increased pollution in metropolitan areas may also play a role in risk of developing NSCLC (Ahn et al., 2013). Due to this variability, geographic location can also influence risk of NSCLC incidence in AAs, CAs, and HAs (Griffiths et al., 2014).

#### 4.2.3 Comorbidities

Comorbidities are another lifestyle factor that may influence NSCLC risk, incidence, and adverse health outcomes in NSCLC patients. Several comorbidities associated with worse survival outcomes in NSCLC patients include myocardial infarction, congestive heart failure, diabetes with and without complications, and chronic pulmonary disease (Griffiths et al., 2014). In a study conducted by Ahn et al. (2013), the Charlson Comorbidity Index was used to evaluate the influence of medical comorbidities on NSCLC outcomes by stage. The Charlson Comorbidity Index is a measure of the degree of association between 19 different chronic diseases including the examples mentioned above, and their association with mortality (Griffiths et al., 2014).

Out of the 19 chronic diseases, the most prevalent comorbidities observed in NSCLC included chronic obstructive pulmonary disease (COPD), coronary artery disease, diabetes, a prior lung cancer tumor, chronic heart failure, peripheral vascular failure, and cerebrovascular diseases respectively. The findings suggested that patients diagnosed with specific NSCLC tumor histological subtypes were also associated concurrently with having one or more comorbidities. Generally, squamous cell carcinoma tumor subtypes were associated with being males, older age, and at earlier stage at diagnosis (Stage I and II). Furthermore, patients without comorbidities had a significantly higher diagnosis rate at earlier stages of NSCLC in comparison to patients with comorbidities, suggesting that comorbidities also contribute to survival outcomes of NSCLC in patients and potentially exacerbate disease burden leading to higher rates of mortality (Sigel et al., 2012). Another study found that patients with several different cancer types including lung cancer, were also likely to have undiagnosed diabetes. The majority of these patients were found to have limited access to healthcare, were less likely to see a healthcare provider on a routine basis, and had more advanced stages of cancer (Islam et al., 2015).

HIV was also highlighted as an independent risk factor for incident lung cancer, as it is considered the most common cancer diagnosed among patients that also have HIV (Sigel et al., 2012; Ahn et al., 2013; Griffiths et al., 2014). The study suggested that this higher incidence rate of lung cancer among HIV patients is potentially driven by deficiencies in the physiological functions of the immune system as a result of HIV incidence (Williams et al., 2013). Moreover, in terms of NSCLC outcomes in different racial and ethnic populations, Williams et al. (2013) conducted a study to investigate the role of comorbidities in contributing to racial differences in receiving early-stage NSCLC in US veteran lung cancer patients. The results suggested that black patients were more likely to have higher rates of hypertension, liver disease, renal disease, history of drug abuse, but a lower rate of respiratory diseases in comparison to Caucasian patients. Despite this, there was no significant differences between black and Caucasian patients with specific comorbidities and receiving surgery for clinical interventions in early stage NSCLC (Mina et al., 2012). However, another study found that AA patients were more likely to develop COPD and lung cancer than any other racial or ethnic group (Mina et al., 2012). Respiratory comorbidities such as COPD can also be used as a determinant for prognosis and course of treatment in NSCLC patients (Mina et al., 2012). Other studies have also observed associations between obesity and lung cancer incidence, with notable findings in associations within AA women regarding increased incidence of lung cancer as a result of obesity, as well as increased mortality (Bethea et al., 2013; Cohen et al., 2014; Putila and Guo, 2014; Campbell et al., 2017).

Future studies should also investigate the lower incidence rates of respiratory diseases in AA patients, as this can help determine which comorbidities are likely associated with increased risk and burden of NSCLC in AA patients, as well as other racial and ethnic groups. Taken together, this suggests that comorbidities are also potentially an influential factor to consider in NSCLC incidence and mortality and should be another parameter considered when measuring NSCLC risk and screening for lung cancer in diverse patient populations.

### 4.3 Biomarker discovery

Genetic mutations and variants have significant clinical value. Several studies in the present literature review highlighted research investigating the use of genetic testing to predict whether a patient will respond to certain treatment types, such as Tyrosine Kinase Inhibitors, and other therapeutic agents. Harboring certain mutations is an important factor to consider when determining course of treatment due to possible effects on sensitivity, or lack thereof, to certain drugs used to target the effects of specific genetic alterations in NSCLC. Additionally, these studies can also be used to discover biomarkers that can be utilized as diagnostic and prognostic tools to promote early detection and improve survival outcome in NSCLC patients. In an additional study observing the frequency of somatic mutations in LUAD and SCC subtypes, Campbell et al. (2017) suggested that these investigations can potentially reveal notable associations between specific alterations within different histological subtypes, which can aid in the discovery of targeted therapies that can work for specific NSCLC tumor types. Some of the genes most notably detected in both LUAD and SCC tumor subtypes include *TP53*, *RB1*, *ARID1A*, *CDKN2A*, *PIK3CA*, and *NFL*. Of these gene, *TP53*, *CDKN2A*, and *PIK3CA* were found significantly in SCC tumor subtypes (*p* < 0.01). Additionally, gene mutations that were detected significantly in other cancer types including colorectal cancer (CRC) and glioblastoma (GBM) were also found at significant rates in LUAD (Yasuda et al., 2013).

Current clinical TKIs that target *EGFR* mutations include erlotinib, getfitnib, and afatinib (Maemondo et al., 2010; Zhou et al., 2011; Sequist et al., 2013; Greathouse et al., 2018; Lusk et al., 2019b). As mentioned previously, studies have shown that identifying the type of *EGFR* mutation a patient harbors in their NSCLC tumors may have significant clinical value in predicating response. For example, insertion mutations of *EGFR* exon 20 are non-responsive to TKIs that target *EGFR* mutations. Additionally, there are several markers associated with smoking that may also be used to assess NSCLC risk and incidence in diverse patient populations. This includes *TP53*, which has been identified as being involved in changes that occur in the lung microbiome during transition from a normal to cancerous state (Greathouse et al., 2018) and imaging markers which include inflammatory proteins and cytokine levels (Pine et al., 2016; Meaney et al., 2019), ^115^.

### 4.4 Limitations

Limitations in cohort design make it increasingly difficult to replicate and produce racially/ethnically diverse studies that can establish the role of genetics and environment when identifying driving factors in NSCLC risk/incidence/survival. Although genetic differences have been found at the ancestry level, including the discovery of novel susceptibility alleles and associations among several mutant variants and risk, prognosis, and treatment response, the lack of significant differences at an individual level with respect to race and ethnicity suggests that observed gene specific mutational frequencies and other genomic differences are possibly driven by environmental factors, and genetic ancestry may be a secondary factor compared to environment.

Further research is necessary to assess and compare the impact of genetic and environmental factors in NSCLC. Having a large racially/ethnically diverse cohort that is representative of a wide range of SES, geographic location types, comorbidities, occupations, level of education, and lifestyle factors including diet, exercise, smoking status, as well as other behaviors and habits can improve our understanding of the associations between these characteristics and assist in identifying other associations between environment and genetics that contribute to NSCLC risk, incidence, and survival.

The studies included in this review have demonstrated that lung cancer has a heterogeneous tumor biology and suggest that mutational analyses and genomic profiling may have clinical value with regard to caring for patients with NSCLC. Understanding the implications of different genetic mutations can support the efficacy of therapeutic drugs and treatment methods in diverse populations.

## 5 Conclusion

In conclusion, racially and ethnically diverse cohort studies can reveal differences in NSCLC disease risk, incidence, and survival outcomes among subgroups within the population. This literature review suggests that there are no significant differences between the mutational profiles of specific genetic mutations that occur in NSCLC among AA, CA, LA, HA populations. Findings also suggest that differences in NSCLC risk, incidence, and survival in racial/ethnic populations may more likely be attributed to general lifestyle, behavioral, and environmental factors which can also influence genetic changes leading to NSCLC, thereby suggesting that these factors may potentially have a greater impact on NSCLC incidence.

Mutational analyses and genomic profiling may have significant clinical value in screening for and treating NSCLC. Understanding the implications of different genetic mutations and gene variants revealed through ancestry level studies can lead to the development of diagnostic and prognostic biomarkers that improve accuracy and specificity of detection of lung cancer at earlier stages, as well as therapeutic methods, and other effective treatment options especially in patients who exhibit lack of sensitivity and response to specific drugs. Future studies should continue to focus on investigating lifestyle/behavioral habits, as well as population genetics, to more fully elucidate the contributors (and interactions) that influence NSCLC risk and incidence, and ultimately reduce mortality rates, address healthcare disparities, and improve overall survival outcomes in high-risk populations.

## Data Availability

The original contributions presented in the study are included in the article/Supplementary Material, further inquiries can be directed to the corresponding author.

## Abbreviations

AA, African American; CA, Caucasian American; HA, Hispanic American; LA, Latino American; AFR, African Ancestry; EUR, European Ancestry; NAT, Native American Ancestry; LUAD, Lung Adenocarcinoma; SCC, Squamous Cell Carcinoma; NSCLC, Non-Small Cell Lung Cancer; Ref, Reference Value; NS, Never Smokers; FS, Former Smokers; CS, Current Smokers; ES, Ever Smokers; CPD, Cigarettes Per Day; M, Mutant; WT, Wildtype.
