# Supplementary material for: A systematic review of genetic ancestry as a risk factor for incidence of non-small cell lung cancer in the US
Source: Front Genet. 2023 Apr 4;14:1141058. doi: 10.3389/fgene.2023.1141058 (PMC10110850; doi:10.3389/fgene.2023.1141058)
Supplement: Supplementary file 1 [file Table1.docx]

**Supplementary Table 1:** Literature Review Summary of Results Table

| Reference | Study Aim | Subgroup | Key Findings |
| --- | --- | --- | --- |
| Yamaguchi et. al, 2013 | To analyze the frequency of somatic mutations in NSCLC among a patient cohort with diverse clinicopathological characteristics including race, gender, age, and tumor histological subtype. | CA AA LA | There were no significant differences in the mutational status of EGFR, KRAS, and ALK Translocations in White, Black, and Hispanic/Latino patients. |
| Campbell et. al, 2017 | To analyze different somatic mutations in black and white NSCLC patients to determine if there were any differences in mutational frequencies of oncogenic drivers commonly identified in NSCLC. | AA CA | There were no significant differences in the mutational status of EGFR, KRAS, and ALK Translocations in White, Black, and Hispanic/Latino patients. |
| Jones et. al, 2017 | To supplement existing evidence by validating established germline genetic variants associated with lung cancer in other races in addition to discovering new variants that are associated with lung cancer survival in AAs. | AA CA | Several variants were detected in this study, although only one, rs1878022, was significantly associated with African Ancestry and an increased risk of mortality. |
| Oak et. al, 2020 | To identify pathogenic and cancer predisposing variants using genetic ancestry analysis methods in NSCLC patients with different ancestry backgrounds. | AFR  EUR  AMR | In African Ancestry samples, BRCA2 was significantly associated with SCC. Only one European Ancestry sample was identified as a BRCA2 carrier. No significant associations were found in Native/Latin American Ancestry samples. |
| Lusk et. al, 2019 | To characterize the mutational profile and mutational frequency of different driver genes associated with NSCLC incidence in AAs. | AA  CA | Successfully identified 77 different driver mutations, with 50 mutually exclusive mutations defined as having detrimental effects in several different genes. |
| Reinersman et. al, 2011 | To broaden existing evidence of previous studies by investigating the rate of EGFR and KRAS mutations in AA patients diagnosed with the LUAD subtype of NSCLC. | AA CA | Study did not find any differences in the mutational frequency of EGFR in AAs and CAs in NSCLC among these two groups. However, the mutational frequency of KRAS was significantly different. |
| Araujo et. al, 2015 | To examine several different gene mutations associated with NSCLC in AAs and CAs to determine whether there are any differences in mutational profiles among both groups | AA CA | Mutational frequency and survival were not significantly different among AA and CA NSCLC patients. Although the overall survival of the AA cohort was higher in patients harboring EGFR mutations. |
| Steuer et. al, 2016 | To explore the differential effects of genetic alterations associated with NSCLC LUAD subtype in AAs, CAs, and LAs | AA CA Latino | Overall, there were no statistically significant differences in the mutational frequency of any of the oncogenic drivers tested in AAs, CAs, and LAs (p = 0.149) |
| Bauml et. al, 2013 | To investigate whether there were any differences in the mutational frequencies of EGFR and KRAS mutations across different racial and ethnic groups (including HAs, AAs, and Cas). | AA CA Hispanic | When comparing AAs & CAs, there were no significant differences found in the mutational frequency of KRAS. Additionally, the mutational frequency of EGFR was significantly associated with race (p < 0.001). |

**TS1 |** Continued

| Reference | Study Aim | Subgroup | Key Findings |
| --- | --- | --- | --- |
| Rodriguez et. al, 2019 | This study aims to analyze genetic mutations associated with the NSCLC histological subtype LUAD in AAs (i.e. KRAS, EGFR, TP53). | AA CA | There were no statistically significant differences among AAs and CAs and mutational frequency of driver genes in NSCLC found in this study |
| Arcila et. al, 2013 | To examine the frequency of the EGFR exon 20 insertion mutation in NSCLC. | CA | When compared to patients that did not have EGFR exon 20 insertions, these mutations were more common among never smokers (statistically significant). |
| Sholl et. al, 2015 | To analyze the genetic mutational profiles of NSCLC patients diagnosed with the LUAD histology, including KRAS, EGFR, ALK Translocations, ERBB2, and MET. | AA  CA | There were no significant differences in mutational frequencies among black and white patients with LUAD (NSCLC). |
| Cote et. al, 2011 | To investigate the frequency and features associated with EGFR mutations in NSCLC cases among AAs and CAs to determine whether the mutational frequency of EGFR varies between AAs and CAs. | AA CA | There were no significant differences in the mutational frequencies of EGFR among black and white NSCLC patients. The only difference, although not significant, was AAs being older at time of diagnosis. |
| Bollig-Fischer et. al, 2015 | To quantitatively assess the mutational frequencies of several oncogenic drivers associated with NSCLC in a AA cohort. | AA CA | There were no statistically significant differences in the mutational frequencies of mutually exclusive point mutations among AAs and CAs. |
| Cheng et. al, 2019 | To investigate survival outcomes of NSCLC in AA versus Non-AA patients (not specified) with or without EGFR mutations. | AA Non-AA (NOS) | AA patients with EGFR-positive tumors have a lower survival rate in comparison to Non-AA patients, which also impacted treatment options. |
| Arauz et. al, 2020 | To characterize the mutational profile and mutational frequency of different driver genes associated with NSCLC incidence in AAs. | AA  CA | The results also suggest that the STK11 was significantly mutated at a higher frequency in AA patients than in white patients (p = 0.002) |
| Reckamp et. al, 2021 | To utilize genetic mutational analyses to identify clinical characteristics associated with high risk of developing mutations, and subsequently NSCLC. | AFR  EUR  AMR  HA | Germline point mutations including TP53, EGFR, and BRCA2 are potentially associated with earlier diagnosis of NSCLC, specifically in the LUAD histology. |
| Kytola et. al, 2017 | To characterize the mutational profile and mutational frequency of different driver genes associated with NSCLC incidence in AAs, driven by smoking habits. | AA  CA  LA | Cancers associated with smoking were associated with a higher mutational frequency, including TP53 and the BRCA2 genes in AAs. |

**TS1** | Continued

| Reference | Study Aim | Subgroup | | Key Findings | |  |
| --- | --- | --- | --- | --- | --- | --- |
| Villaruz et. al, 2015 | To characterize the mutational profile and mutational frequency of BRAF associated with NSCLC incidence and survival. | | AA  CA | | There were no statistically significant differences in the mutational frequency of BRAF among AAs and CAs. | |
| Al-Ahmadi et. al, 2020 | To characterize the mutational profile and mutational frequency of different driver genes associated with NSCLC. | | AA  CA | | There were differences in the mutational frequencies of several driver genes among AAs and CAs although not statistically significant. | |
| Vigneswaran et. al, 2016 | To characterize the mutational profile and mutational frequency of different driver genes associated with NSCLC incidence in using Next Generation Sequencing | | AA  CA | | There were no statistically significant differences in the mutational frequencies of mutually exclusive point mutations among AAs and CAs. | |
| Gill et. al, 2011 | To characterize the mutational profile and mutational frequency of LKB1/STK11 in CAs and AAs. | | AA  CA | | There were no statistically significant differences in the mutational frequencies among AAs and CAs, although the frequency of homozygous deletion of LKB1/STK11 was higher in CAs than in AAs. | |
| Sinha et. al, 2020 | To analyze the frequency of homologous recombination deficiency in genes characterized as pathogenic variants associated with NSCLC incidence in AAs and CAs. | | AA  CA | | In AA NSCLC Patients diagnosed with the SCC subtype, there was a higher frequency of genomic instability and homologous recombination deficiency in comparison to CAs. | |
| Jones et. al, 2019 | To identify novel variants associated with NSCLC risk in AAs using pleiotropic analyses. | | AFR  EUR | | Several novel variants associated with increased risk of NSCLC incidence were identified in individuals with African Ancestry. These three novel variants include: rs336958, rs7186207, and rs11658063. | |
| Zanetti et. al, 2016 | A GWAS of lung cancer utilizing ancestry mapping to identify SNPs associated with NSCLC. | | AFR  EUR | | Several novel variants associated with increased risk of NSCLC incidence were identified in individuals with African Ancestry. These three novel variants include: rs55781567, rs3019885, and rs6580649. | |
| Schenk et. al, 2020 | A GWAS of lung cancer utilizing ancestry mapping to identify somatic mutations associated with NSCLC. | | AFR  EUR | | These findings suggest that African Ancestry and European Ancestry influence somatic mutational frequencies in the LUAD histology. | |
| Adib et. al, 2022 | A GWAS of lung cancer utilizing ancestry mapping to identify somatic mutations associated with NSCLC. | | AFR  EUR | | These findings suggest that ancestry has a significant influence on genomic alterations associated with NSCLC incidence. | |
| Mitchell et. Al, 2019 | A GWAS of lung cancer utilizing ancestry mapping to identify somatic mutations associated with NSCLC risk and survival in AAs. | | WAA  EUR  AMR | | These findings suggest that increased West African Ancestry is associated with having an increased risk of NSCLC incidence in AA men (p = 0.001) although there was no significant association identified in AA women. | |
| Jones et. al, 2018 | A GWAS of lung cancer utilizing ancestry mapping to identify somatic mutations associated with NSCLC risk and survival in AAs. | | AFR | | African ancestry was not significantly associated with increased or decreased NSCLC risk, incidence, and survival. | |
| Byun et. al, 2018 | A GWAS of lung cancer utilizing ancestry mapping and family history to identify associations with NSCLC risk. | | EUR | | Several novel variants were identified confirming increased an association with increased risk of NSCLC incidence in the SCC histology. | |

**TS1** | Continued

| Reference | Study Aim | Subgroup | Key Findings |
| --- | --- | --- | --- |
| Ji et. al, 2020 | A GWAS of lung cancer utilizing ancestry mapping to identify associations with NSCLC risk. | EUR | Results suggest that the ATM L230F gene mutation (variant rs56009889) was significantly associated with LUAD NSCLC incidence. |
| Zhou et. al, 2017 | A GWAS of lung cancer utilizing ancestry mapping to identify associations with NSCLC risk. | EUR | The KANSARL gene is the first predisposing fusion gene associated with lung cancer incidence identified in individuals with European Ancestry. |
| Orloff et. al, 2012 | A GWAS of lung cancer utilizing ancestry mapping to identify somatic mutations associated with NSCLC risk and survival in AAs. | EUR | These findings suggest that ancestry has a significant influence on genomic alterations associated with NSCLC incidence. |
| Zingone et. al, 2021 | A GWAS of lung cancer utilizing ancestry mapping to characterize the mechanism of alternative polyadenylation sites in the genome associated with NSCLC risk. | AFR  EUR | These findings suggest that alternative polyadenylation is significantly more likely to occur in AA NSCLC patients in comparison to CA NSCLC patients. |
| Li et. al, 2018 | A GWAS of lung cancer utilizing ancestry mapping to identify somatic mutations associated with NSCLC risk and survival in individuals with European Ancestry. | EUR | Several novel variants were identified confirming increased an association with increased risk of NSCLC incidence and smoking: rs6441286, and rs17723637. |
| Saeed et. al, 2012 | A GWAS of lung cancer utilizing ancestry mapping to identify associations with NSCLC risk and survival. | HA  AMR | Hispanic patients and Native American ancestry was significantly associated with increased overall survival associated with all NSCLC histological subtypes in comparison to AAs and AFR ancestry.. |
| Gimbrone et. al, 2017 | A GWAS of lung cancer utilizing ancestry mapping to identify somatic mutations associated with NSCLC risk and survival in Hispanic/Latino Americans. | AMR  HA  NHW | Hispanic Ancestry, although not significant, was associated with an increased rate of TP53 mutations (p=0.009). Additionally, in comparison to NHW patients, EGFR, KRAS, and STK11 occurred at a lower mutational frequency in Hispanic patients. |
